# Supplementary material for: Ultraviolet-Sensitive Properties of Graphene Nanofriction
Source: Nanomaterials (Basel). 2022 Dec 15;12(24):4462. doi: 10.3390/nano12244462 (PMC9785420; doi:10.3390/nano12244462)
Supplement: Supplementary file 1 [file nanomaterials-12-04462-s001.zip › nanomaterials-2079475-supplementary.pdf]

nanomaterials

Supplementary Materials

Ultraviolet-Sensitive Properties of Graphene Nanofriction

Gaolong Dong, Shuyang Ding * and Yitian Peng

College of Mechanical Engineering, Donghua University, Shanghai 201620, China
* Correspondence: shuyangding@dhu.edu.cn

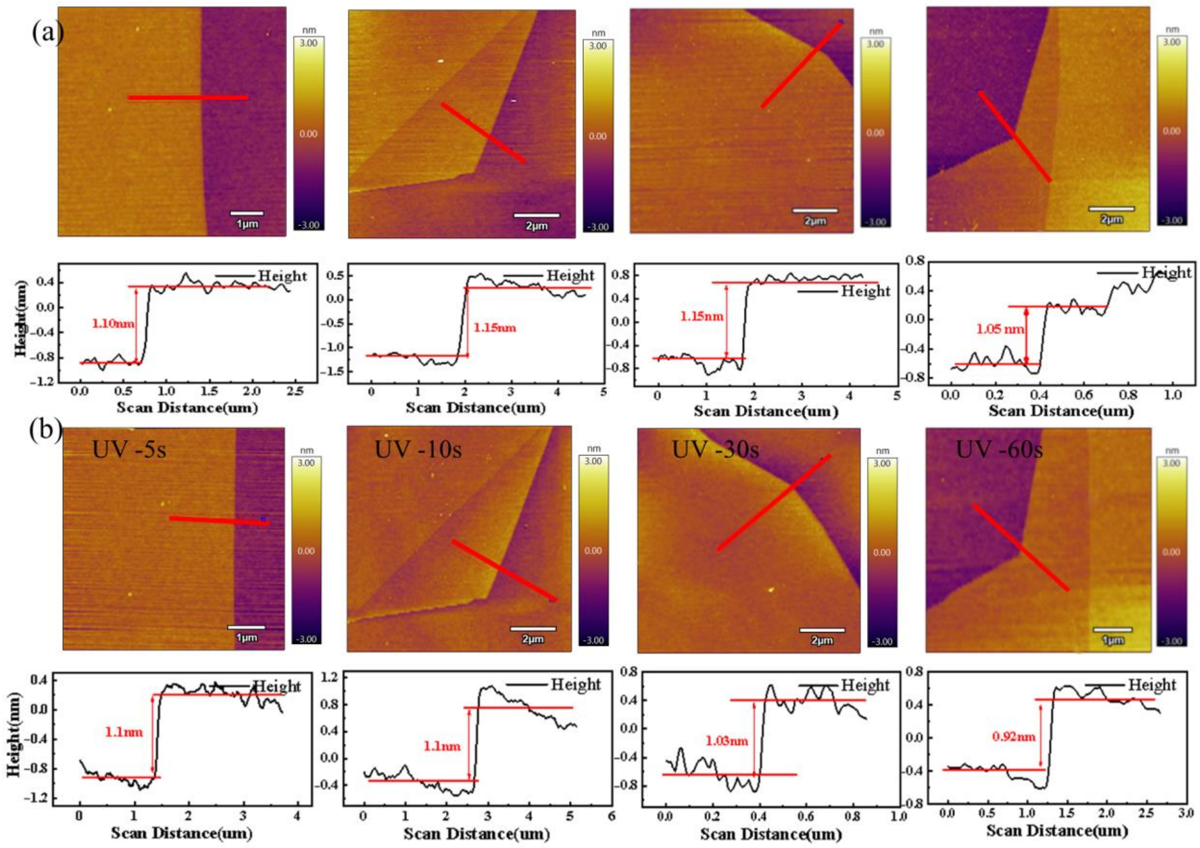

Figure S1. AFM morphologies and height diagrams of graphene with thicknesses of 1 nm under UV vacuum irradiation at different times(a) Before UV vacuum irradiation (b) after UV vacuum irradiation.

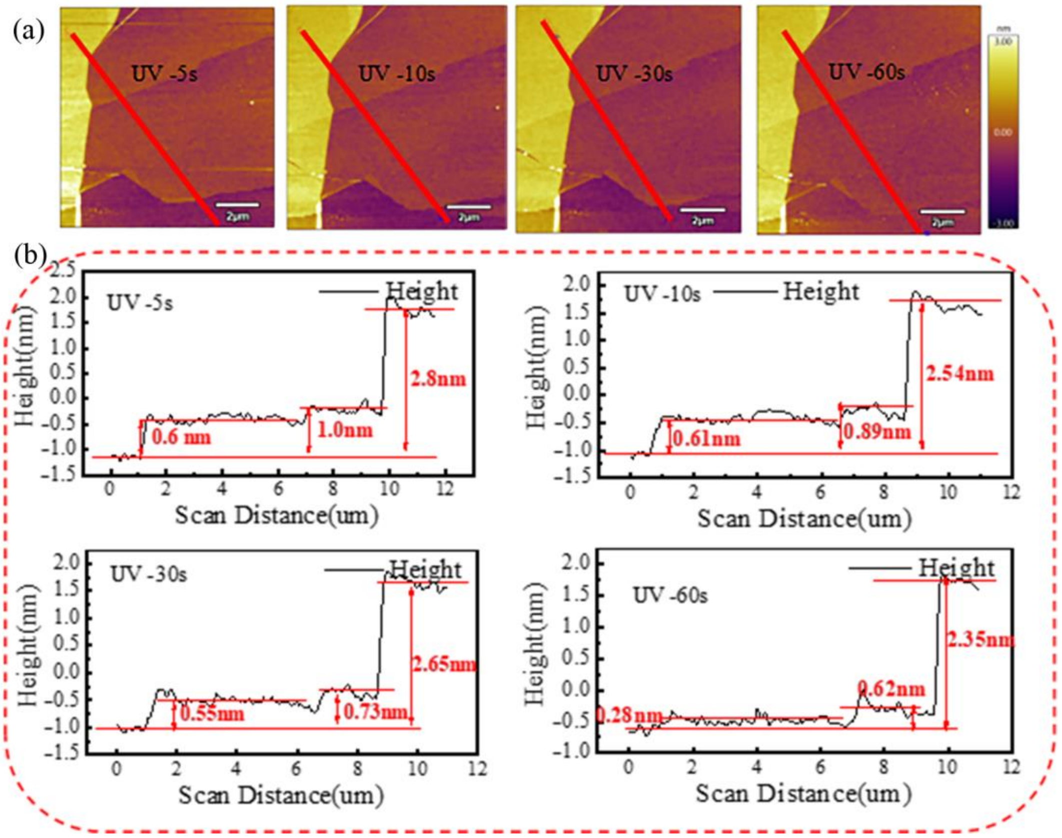

Figure S2. Single sample accumulation experiment (a) AFM morphology (b) indicates the height map in (a) corresponding to the red cut-off line out of the graph.

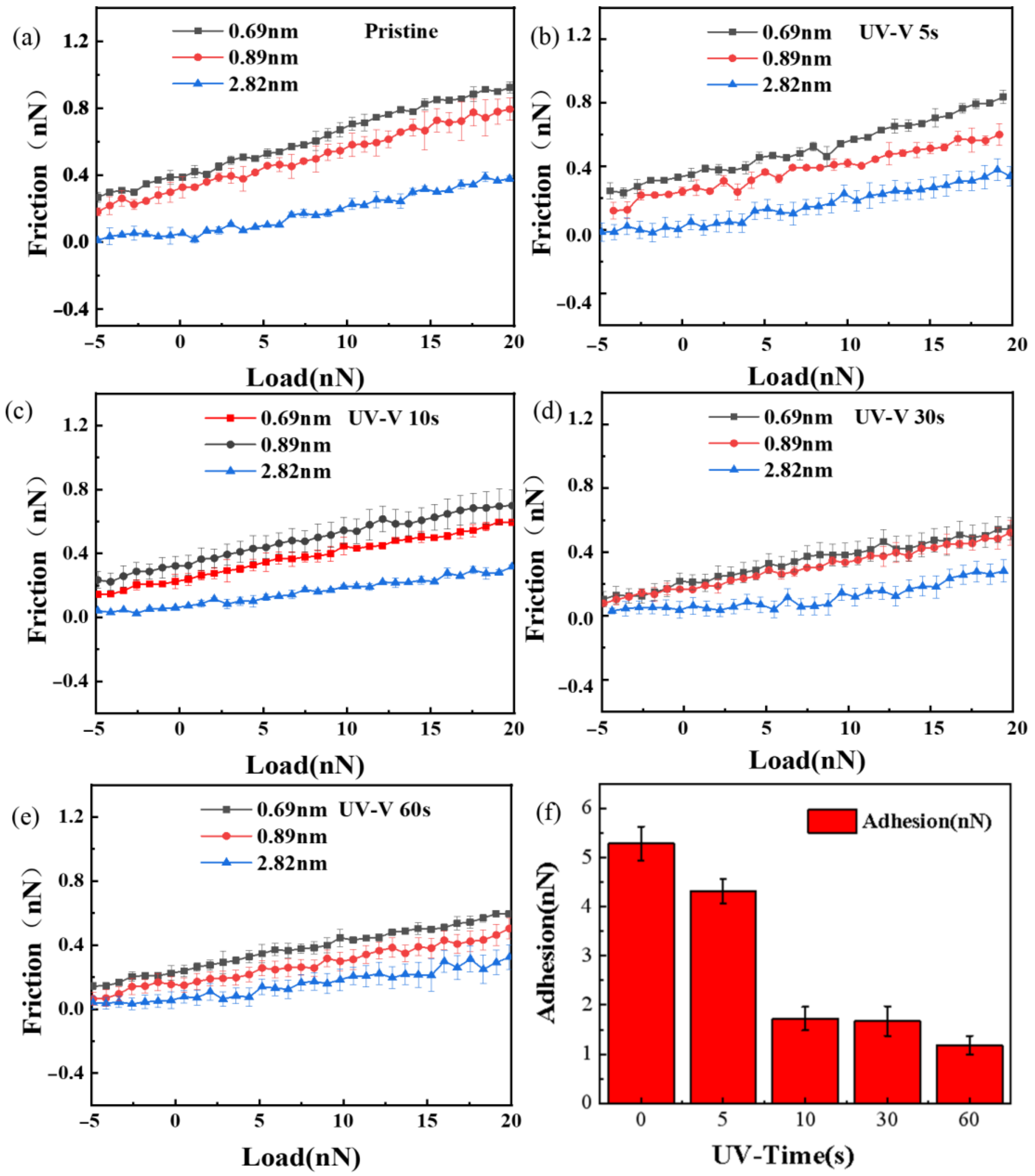

Figure S3: (a-e) the friction forces of graphene with thicknesses of 0.69 nm, 0.89 nm and 2.82 nm after UV vacuum irradiation for 0s, 5s, 10s, 30s and 60s, respectively. (f) indicates the adhesion forces (at 10 nN load) of 0.69 nm graphene with different UV vacuum irradiation times.

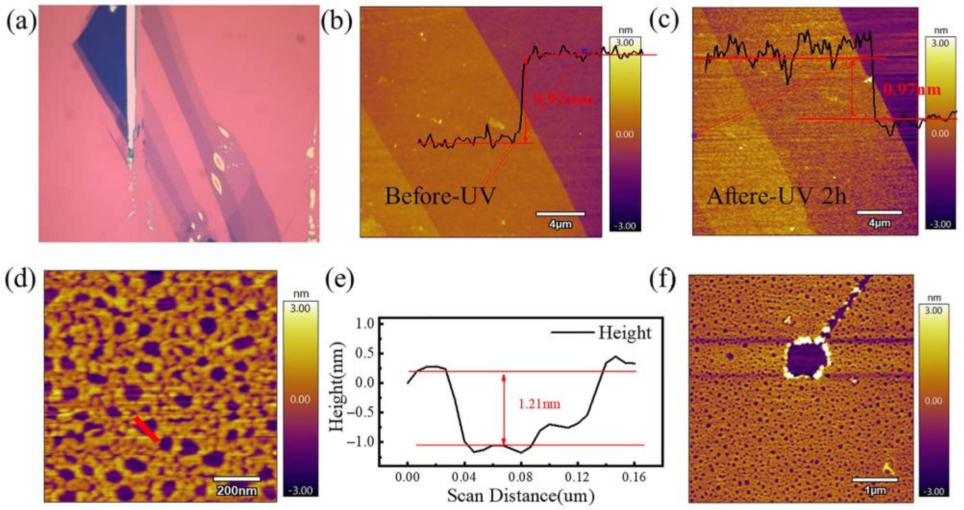

Figure S4. (a) Optical microscope images; (b) AFM morphology before ultraviolet vacuum irradiation; (c) AFM morphology after UV vacuum irradiation for 2h; (d) Microscopic defects of single-layer graphene (1μm×1μm) after 1min UV irradiation; (e) is (d) the height of the position of the colored transversal; (f) The structure of single-layer graphene was damaged for a long time under UV irradiation on the 40nN loading surface.

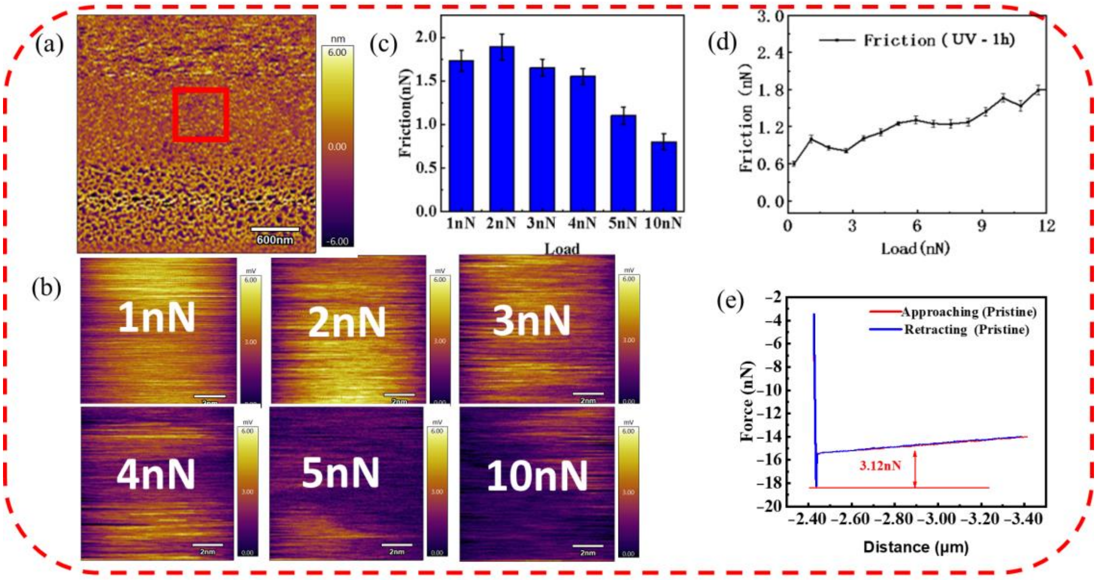

Figure S5. (a) Microstructure of single-layer graphene after 1h UV vacuum irradiation; (b) is the constant load friction diagram in the red box in figure (a); (c) represents the surface sweep friction corresponding to (b); (d) Unloading tests on defective graphene; (e) represents the adhesion test at the defect graphene.

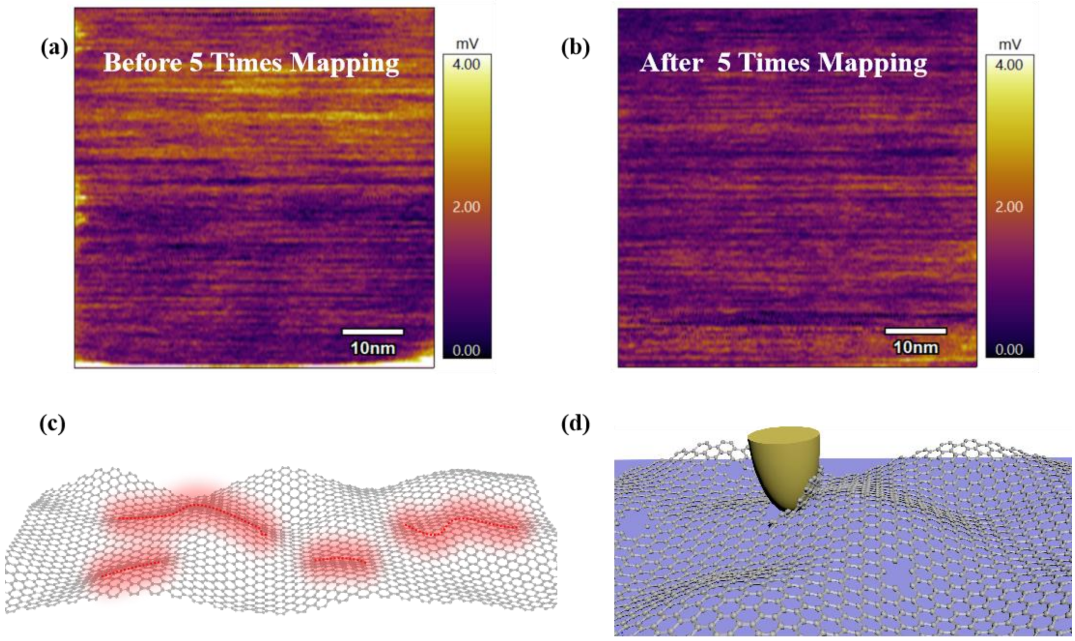

Figure S6. (a) and (b) represents the friction diagram of graphene with structural defects before and after repeated friction for 5 times under the same load at the same position after UV vacuum irradiation for 1h. (c) Schematic diagram of graphene-producing structural defects; (d) Indicate the transfer process of the graphene fragment to the tip of the needle under a certain shear force.
